# Drug-Coated Balloons in All-Comer Population—Are We There Yet?

**DOI:** 10.3390/jcm14103608

**Published:** 2025-05-21

**Authors:** Florin-Leontin Lazar, Horea Laurentiu Onea, Calin Homorodean, Ioan Cornel Bitea, Diana Raluca Lazar, Mihai Claudiu Ober, Dan Tataru, Maria Olinic, Mihail Spinu, Teodor Paul Kacso, Dan-Mircea Olinic

**Affiliations:** 1 Medical Clinic Number 1, 4th Department of Internal Medicine, “Iuliu Haţieganu” University of Medicine and Pharmacy, 400012 Cluj-Napoca, Romania; lazar.leontin@yahoo.com (F.-L.L.); tataru.cardio@gmail.com (D.T.); maria.olinic@yahoo.com (M.O.); spinu_mihai@yahoo.com (M.S.); danolinic@gmail.com (D.-M.O.); 2 County Emergency Hospital, 550245 Sibiu, Romania; cornelioanbitea@yahoo.com; 3 Department of Interventional Cardiology, Cluj County Emergency Hospital, 400006 Cluj-Napoca, Romania; mihai_ober@yahoo.com (M.C.O.); teokacso@gmail.com (T.P.K.); 4 10th Department of Oncology, “Iuliu Hatieganu” University of Medicine and Pharmacy, 400012 Cluj-Napoca, Romania; lazardianaraluca@gmail.com

**Keywords:** drug-coated balloon, drug-eluting stent, coronary artery disease, percutaneous coronary interventions

## Abstract

With the advancement of interventional coronary procedures, drug-coated balloons have become an increasingly common alternative to drug-eluting stents in the treatment of various lesions. This paradigm shift stems from several advantages that DCBs entail, including a reduction in stent length burden, the possibility of late vessel positive remodeling, and the preservation of bifurcation anatomy. Conversely, several studies compared the efficacy of DCB treatment to stents or POBA in various scenarios. In this review, we will discuss the areas in which a DCB can be of paramount importance. We will begin by examining the role of DCBs in in-stent restenosis, for which the current practice guidelines do not clearly state the role of this technology, as opposed to the previous ones, in which it was mentioned as a first-line armamentarium. We will then discuss the indications and advantages of using DCBs in de novo lesions, concerning both small and large vessels, with growing emphasis on diffuse lesions. Lastly, we will address the current data on the use of DCBs in special scenarios such as the treatment of chronic total occlusion and left main and bifurcation lesions, without forgetting the primordial role of drug-eluting stents in all these lesions.

## 1. Introduction

**Figure 1:**
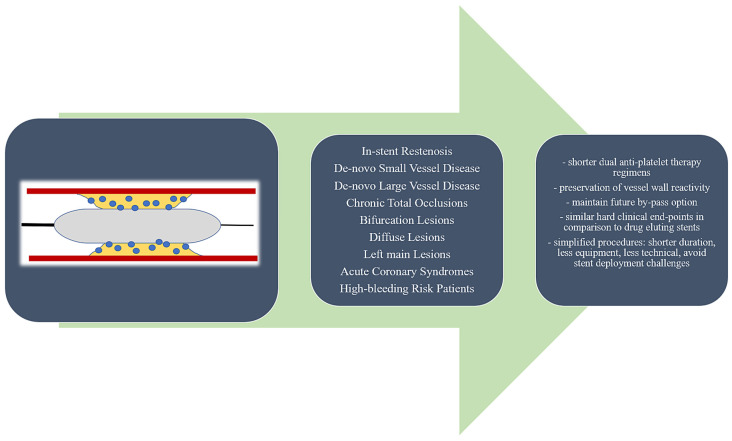
Performance of drug-coated balloons in different complex settings.

Drug-coated balloons (DCBs) have become a valid alternative to drug-eluting stents (DESs) for the treatment of in-stent restenosis (ISR) and de novo lesions in small vessel disease. Although initially it was considered that the key to obtaining a good result after a DCB percutaneous coronary intervention (PCI) was the selection of patients and lesions, later on it became obvious that the more complex the lesion is, the more beneficial the limitation of metallic prosthetics implantation is. As a result, in recent years, multiple trials and registries have reported promising results for their use in more complex settings as well, such as large vessel de novo lesions, bifurcations (including left main lesions), acute coronary syndromes, diabetic patients with multivessel disease, or high bleeding patients [1]. As calcific lesions, long stents, stent malapposition/underexpansion, and premature dual antiplatelet therapy (DAPT) discontinuation have been identified as predictors of stent failure [2,3], the use of a DCB in such cases appears well reasoned. The purpose of the present paper is to review the most recent data on the use of DCBs in complex lesions and patients, as it is our strong belief that DCBs might have the potential to become a first-line armamentarium in treating complex lesions, with bailout stenting in case of suboptimal results. Figure 1 summarizes the complex settings in which DCBs have been tested with promising results.

One of the major objectives of this paper is to individually analyze all of the clinical and angiographic scenarios where evidence suggests that DCBs might be beneficial and corroborate this information in a possible algorithm that might help physicians to better understand when, how, and why to choose a DCB approach, as can be seen in the graphical abstract.

## 2. Dcb in In-Stent Restenosis

### 2.1. Guideline Recommendations

In-stent restenosis represents the first setting for which DCBs were tested, used, and granted a class I indication in the European Society of Cardiology and European Association for Cardio-Thoracic Surgery (ESC/EACTS) Guidelines in 2019 [4]. Surprisingly, in the latest ESC Guidelines [5], this indication was changed and DES treatment for DES-ISR is now recommended over a DCB. However, this document did not mention the role and indication for DCB use in these lesions despite the fact that multiple trials and meta-analyses reported no significant differences in clinical endpoints between the two strategies for treating DES ISR at mid- and long-term follow-up [5,6]. What is more, DCB treatment for bare-metal stent ISR was similar to DES treatment in terms of safety and efficiency [7], again without a clear class indication for its use in this setting.

### 2.2. Evidence from Major Trials

These surprising and somehow unclear new guideline indications for the use of DCBs in ISR should therefore lead to further trials on this topic, as there are robust historical data to support the use of DCBs for this type of lesion. First of all, the 10-year follow-up of the ISAR-DESIRE 3 trial patients compared the composite of cardiac death, target vessel myocardial infarction, target lesion thrombosis, and target lesion revascularization between three different strategies used for treating DES-ISR: plain balloon (PB), paclitaxel-coated balloon (PCB), and paclitaxel-eluting stent (PES) [8]. The primary endpoint occurred in 90 patients (72.0%) assigned to PB, 70 patients (55.9%) assigned to PCB, and 72 patients (62.4%) assigned to PES (*p* < 0.001), with no significant differences between DCB and DES. What is more, an excess of death and cardiac death associated with stents compared with DCB was observed within 5 years after PCI, though 10-year differences did not reach the threshold of statistical significance [8]. These results are consistent with previously published results at 3-year follow-up of the same study, in which any limus-eluting stent (biolimus, zotarolimus, everolimus, or sirolimus) restenosis treatment with either DCB or DES had similar target lesion revascularization (TLR) rates, while DCB treatment in this setting was associated with lower risk of death [9].

In another important meta-analysis, as well cited in the ESC document as an argument for everolimus-eluting stents (EESs), 962 patients from five trials were included and analyzed [10]. At 1-year follow-up, no statistically significant difference in the risk of target vessel revascularization (TVR) was described between DCB and EES (RR 1.15, 95% CI 0.60–2.19), but TVR was increased with DCB at 3 years (RR 1.87, 95% CI 1.15–3.03), but without any differences in the risk of hard clinical endpoints. What is more, as stated by the authors, the quality of evidence was moderate, which in our opinion should not disannul the good results described in so many previous studies that investigated the role of DCBs in DES ISR.

### 2.3. DES ISR vs. BMS ISR—Same but Different?

As the majority of important studies have demonstrated the non-inferiority of DCB versus DES for the treatment of BMS ISR [8,10,11], there are also multiple studies that have demonstrated the efficacy of this technology in DES ISR as well. In the PEPCAD-DES study, DCB was demonstrated to be a safe and effective method to treat in-stent DES restenosis and net superior to plain old balloon angioplasty (POBA) in terms of TLR (19.4% vs. 36.8%; *p* = 0.046) and major adverse cardiac events (MACEs) (20.8% vs. 52.6%, log-rank *p* = 0.001) [12].

On the other hand, in order to have a clear picture on the best available option to treat ISR we have to highlight several aspects and limitations of the previous studies: first of all, as more than 20 years have passed since the first DESs became available for clinical practice, it is most likely that in present times when dealing with ISR, in most of the cases a DES is involved; secondly, in today’s practice it is unrealistic and without real clinical relevance to compare drug-eluting devices to POBA. Therefore, the results from the DAEDALUS [11] study, which reported inferior effectiveness for DCB angioplasty when compared to repeat DES implantation for DES-ISR, should be kept in mind and further investigated in larger trials. These results were reproduced in another meta-analysis of five randomized clinical trials (RCTs) with about 1193 patients, in which repeat DES implantation was associated with a significant reduction in the term of TLR compared with DCB angioplasty [13]. Some histopathological explanations have been proposed for these findings. First of all, it has been suggested that the tissue leading to DES restenosis might be resistant to antiproliferative drugs, as the smooth muscle cell phenotypes differ from those encountered in restenotic tissue from BMS (more contractile or intermediate with DES, which could lead to antiproliferative drug resistance) [14]. However, in the DAEDALUS study, irrespective of type of stent, TLR was higher in patients with smaller minimal lumen diameter (MLD), suggesting that obtaining an optimal MLD is crucial for increasing the chances of good late outcomes when using a DCB, whereas a suboptimal MLD after lesion preparation should lead the operators to use an everolimus-eluting stent [11,14]. Nevertheless, it should be noted that in the DAEDALUS study, the primary safety point, a composite of all-cause death, myocardial infarction, and target lesion thrombosis at 3 years was numerically lower in the DES-ISR treated with DCB group, suggesting the safety of this approach in DES-ISR as well, as leaving nothing behind reduces the risk of chronic wall injury and inflammation with subsequent risk of late stent thrombosis.

Taking all of this into consideration, using a DCB for in-stent restenosis, both DES and BMS, is still very appealing, despite recent guideline recommendations, which are unclear for the moment. However, some aspects should be taken into consideration when using a DCB for DES-ISR: (a) proper understanding of the ISR mechanism is mandatory, and this can be achieved by using intracoronary imaging; (b) proper lesion preparation, including obtaining an optimal minimal lumen diameter, is also mandatory; and (c) when the previous points are not reached, an everolimus-eluting stent is the more effective option, as recommended in the guidelines, but with a higher (numerically but not statistically significant) risk of late stent thrombosis. Therefore, larger randomized clinical trials with long-term follow-up are still needed in order to better understand the role of DCBs in DES-ISR, which not long ago was a class I level of evidence A indication.

## 3. Dcb in De Novo Small Vessels

While in-stent restenosis is the most studied applicability of the DCB strategy amongst all coronary lesions, regarding de novo lesions, small vessels have the most robust data and it is now clear that DCB angioplasty is a safe and efficient treatment for this type of lesion. However, the current guidelines are still lacking a clear indication for its use in this setting, despite the fact that the most important randomized clinical trials, PICCOLETO II [15,16], BASKET-SMALL2 [17], and RESTORE SVD [18], and meta-analyses have reported good long-term results of this strategy in terms of safety and efficacy, which we will briefly present.

In the PICCOLETO II international trial, 232 patients from five centers with lesions occurring in vessels less than 2.75 mm were randomized to PCI using either DCB or DES, with in-lesion late lumen loss (LLL) at 6 months being the primary endpoint and non-inferiority between the two arms being hypothesized. LLL was significantly lower in the DCB group, while clinical endpoints were similar at 12 months [15]. After 3 years of follow-up, MACEs and acute vessel occlusion occurred more frequently in the DES group, but still with no differences between the groups in terms of hard clinical endpoints [16].

The BASKET-SMALL 2 trial enrolled 758 patients with significant coronary lesions in vessels < 3 mm from 14 centers from Europe and randomized them to either DCB or DES PCI, following them for 3 years for MACE (i.e., cardiac death, non-fatal myocardial infarction, and target-vessel revascularization), all-cause death, probable or definite stent thrombosis, and major bleedings [17]. The authors reported very similar results for both groups for all of the above endpoints, but with numerically (but not statistically significant) lower rates of stent thrombosis and major bleeding in the DCB group after the 3-year period of follow-up. However, these results should be interpreted with caution, as a <3 mm diameter, which represented the inclusion criteria, is too generic and differences between a 2 mm and a greater than 2.5 mm vessel might impact long-term outcomes.

The RESTORE SVD China trial enrolled 230 subjects with lesions occurring in coronaries smaller than 2.75 mm (but >2.25 mm), which were enrolled to either Restore DCB or the RESOLUTE Integrity DES PCI, with 9-month and 1-year angiographic and clinical follow-up being planned [18]. DCB achieved non-inferiority compared with the DES (*p* for non-inferiority < 0.001) in terms of in-segment percentage diameter stenosis, but without any differences in 1-year rates of target lesion failure, with consistent results at 5 years of follow-up.

Recently, a cutting-edge meta-analysis, the ANDROMEDA study, was published. The study included 1154 patients (1360 lesions) from three eligible trials (random allocations of treatments; small vessel disease lesions; treatment with paclitaxel-coated balloons or DES; and clinical follow-up of at least 36 months). Not surprisingly and consistent with the results of another meta-analysis, this study reported a non-significant difference in target lesion failure (TLF) at 3-year follow-up, but with a lower MACE rate compared with DES, due to a lower risk of MI and TVR [19]. Table 1 summarizes the main findings of these major studies.

**Figure 2:**
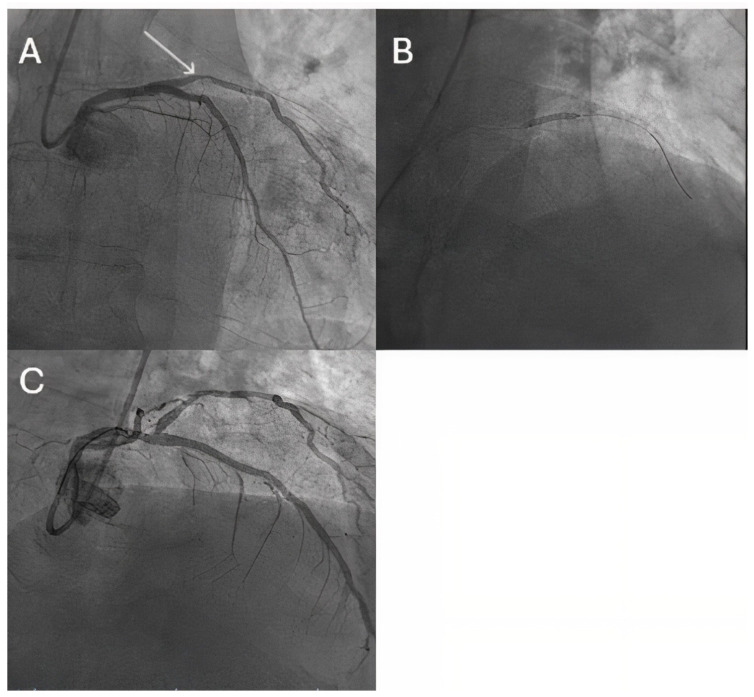
DCB role in small vessel disease. (**A**) Diagnostic angiography revealing 70% stenosis in a 2.5 mm diagonal (white arrow). (**B**) Paclitaxel-coated balloon well expanded in the lesion (after proper lesion preparation). (**C**) Final good angiographic result.

It should be noted that all of these studies investigated the role of paclitaxel-coated balloons, which is known for inducing a late lumen enlargement, as a consequence of its positive vessel remodeling effect, due to its capacity to reach the adventitia; however, lately, more sirolimus-coated balloons have emerged as a promising armamentarium for dealing with small vessel disease, but their role is still to be evaluated [20]. Figure 2 illustrates the role of DCBs in a small diagonal of 2.5 mm, as part of our routine practice.

## 4. Dcb in De Novo Large Vessels

Keeping in mind the good results obtained in de novo lesions occurring in small vessels, the safety and efficacy of DCB in larger than 2.75 mm vessels have become of major interest in recent years, with more and more studies and trials reporting satisfactory results with a “leave nothing behind” strategy. Before presenting these results, there are, however, a few aspects that have to be clarified from the very beginning.

### 4.1. The “Leave Nothing Behind” Concept—Rationale, Advantages, and Pitfalls

The concept of leave nothing behind in large vessels relies on the multiple theoretical advantages of this strategy, which might overcome the limitations of current-generation stents. Therefore, the use of DCB in large vessels might lead to the avoidance of stent malapposition, late strut fracture, and late wall inflammation, restoration of cyclic pulsatility and normal vasomotion, reduction in DAPT duration, facilitation of coronary target lesion re-interventions, and avoidance of full-metal jackets, restoring late by-pass surgery options, which is of great interest, especially in young patients [3]. However, there are multiple pitfalls of this technology that should be taken into consideration, such as the risk of major dissections during predilatation, more recoil compared to metallic stents, which can lead to acute vessel closure, and the lack of evidence from major randomized trials, as opposed to drug-eluting stents, for which there are robust data from multiple important trials that validate their use in this scenario [3].

There are several procedural aspects that are mandatory in order to perform a safe DCB angioplasty in large vessels: (a) Aggressive lesion preparation should always be performed when complex calcific lesions are encountered, even with the risk of obtaining non-flow-limiting dissections, as they are not associated with an increase in adverse events [21]. In order to obtain a good predilatation result, conventional balloons are recommended for non-calcific, simple lesions; however, in calcific lesions, the use of cutting/scoring balloons, as well as a more complex armamentarium such as intravascular lithotripsy, atherectomy (rotational/directional), or laser therapy, is mandatory [3,22]. (b) Imaging should be used in most of the DCB PCI procedures in order to obtain better long-term results, as has been shown by the ULTIMATE III trial, in which IVUS-guided DCB angioplasty was associated with a lower LLL compared with angiography guidance alone (−0.10 ± 0.34 mm vs. 0.03 ± 0.52 mm, mean difference 0.14 mm; 95% CI: 0.02–0.26; *p* = 0.025) [23]. Similarly, optical coherence tomography (OCT) has been shown to improve the outcomes after DCB use, mainly by helping the operators to better understand the plaque morphology and therefore choose adequate predilatation tools, better assess the vessel diameter, lesion length, thrombus identification, and assess post-DCB results in terms of adequate lumen gain, absence of tissue prolapse, significant recoil, or major dissections [24].

### 4.2. Evidence from Major Trials

Starting from these points, in recent years, several studies have demonstrated that good long-term results using DCB are an achievable goal for native large vessels as well. In a recently published large retrospective study, 2857 consecutive patients with 708 lesions treated with paclitaxel DCB only and 704 patients with DES only (matched after propensity score matching out of 2149 lesions) were enrolled and analyzed at 2-year follow-up (25), with clinically driven TLR being the primary endpoint. As lower bleeding rates were observed in the DCB-only group (0.8% vs. 3.0%, *p*  =  0.003) as a result of shorter DAPT regimens, this strategy seems suitable for this type of patient, but with a higher risk of TLR (DCB: 5.5%, sDES: 3.1%, *p*  =  0.028), especially when residual stenosis > 30%, severe calcified lesions, or diabetes are present [25].

**Figure 3:**
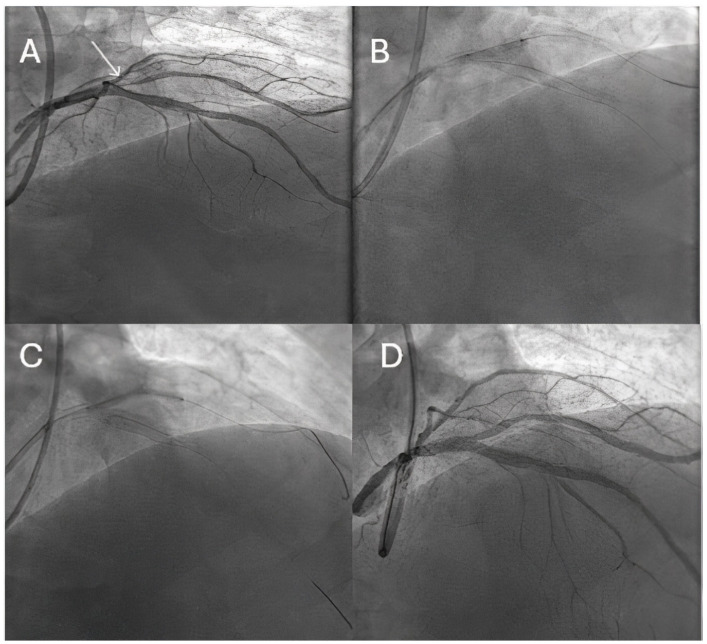
DCB role in large coronary arteries. (**A**) Diagnostic angiography revealing an ostial lesion of a 3.2 mm diagonal (white arrow). (**B**) Predilatation using a scoring balloon of 3.0 mm. (**C**) DCB inflation in the diagonal branch. (**D**) Good final angiographic result.

These results are, however, contradictory to several randomized clinical trials, which showed similar 12-month MACE rates between DCB and DES in large artery disease [26], as well as low two-year follow-up TLF, TLR, and TVR rates of 4.0%, 3.4%, and 4.2%, respectively [27]. In a recently published meta-analysis of seven RCTs, including 816 patients with de novo large vessel disease (422 treated by DCB and 394 by DES), late lumen loss measured at 6–12-month follow-up was statistically significantly lower in the DCB group than in the DES group [28]. What is more, the safety outcomes consisting of cardiac death, myocardial infarction, and target lesion revascularization were similar between the two groups, suggesting that this strategy is safe and efficient in selected patients. In another recent retrospective observational study, Leone PP et al. analyzed the safety and efficacy of DCB alone or as part of a hybrid strategy in combination with DES for treating vessels >3.0 mm with de novo lesions in 93 consecutive patients (100 lesions) [29]. The patients were followed up for a mean period of 350 days, with only a 6.6% rate of TVR and no definite/probable acute vessel occlusion. Despite a long mean lesion length of 45 ± 26 mm (diameter of 3.2 ± 0.3 mm), a DCB-only strategy was performed in 70% of the lesions, with 6% bailout stenting and a hybrid strategy in the rest of the patients, thus considerably reducing the number and length of stents that needed to be implanted. These results emphasize the advantages of this strategy in large vessels as well, as a shorter stent length or no metal prosthesis at all can lead to a shorter dual antiplatelet therapy (DAPT) regimen and maintain vessel wall reactivity and positive remodeling, with a lower risk of neoatherosclerosis. However, as most of the evidence mostly comes from observational studies and meta-analyses, it is clear that the role of DESs in large vessel disease is still in the early stages; however, future data will hopefully clarify the benefits of DCBs in this setting as well. Figure 3 illustrates the role of DCBs in an ostial lesion of a 3.2 mm diagonal, treated by a leave nothing strategy, thus avoiding a true bifurcation technique and obtaining a good angiographic result through a simplified strategy.

## 5. DCB in Diffuse Lesions—Alone or as Part of Hybrid Strategy

As increasing the amount of implanted metal directly correlates with the probability of stent failure, an attractive therapeutical option for diffuse CAD seems to originate from a blended DCB/DES or even a full-DCB strategy. There are currently no dedicated RCTs comparing DCB vs. DES for the treatment of long coronary lesions, although pivotal studies in the field of DCBs (PICCOLETO II, RESTORE SVC, and BASKET-SMALL II [15,16,17,18]) showing promising outcomes in favor of these devices included patients with diffuse lesions. Retrospective cohort studies showed acceptable results for a DCB-only strategy in the treatment of diffuse lesions (TLR 17.7%, MACE 16.5%-paclitaxel-DCB; TLR 12%, MACE 10%-sirolimus DCB) [30,31]. More recently, favorable results were also noted for a hybrid DCB-DES therapy (spontaneous MI rate 2.8%, TVR 5.5%) [32] as well as a full-/hybrid strategy in large coronary arteries (no spontaneous MI, TVR 6.6%) [29].

Costopoulos et al. [33] retrospectively compared DCB vs. DES in long coronary lesions (a hybrid strategy was used in the former group in case of very long lesions, mean length = 67 mm). Similar results were reported in terms of MACE and TLR between the two groups. A recent propensity-matched analysis compared patients with complex coronary artery disease (CAD) (including diffuse lesions) from the EASTBOURNE Registry to patients treated with DES in terms of net adverse cardiovascular events (a composite of death, TLR, non-fatal MI, and major bleedings). During the 1-year follow-up, the primary endpoint occurred less frequently in the DCB group (3.9 vs. 10.5%, *p* = 0.003), mainly due to a lower bleeding risk (*p* = 0.001), even after adjusting for lesion length (HR: 0.23, CI [0.10, 0.52], *p* < 0.001) [34].

Several prospective studies including DCB for diffuse CAD are noteworthy. Buono et al. [35] tested the safety and efficacy of a hybrid DCB-DES therapy including 106 patients with >28 mm lesions. Procedural success was 96% and there were no thrombotic events, while the primary endpoint, a device-oriented composite (cardiac death, target vessel MI, and ischemia-driven TLR) was observed in 3.7% of cases. In the SPARTAN DCB study, the authors compared a DCB-only strategy to DES for stable, de novo CAD (mean lesion length = 26 mm) in 1517 patients. There was a non-significant trend towards better prognosis when using DCB at the 5-year follow-up (*p* = 0.08) [36]. Another head-to-head comparison between DCB (alone or hybrid) and DES was provided by Yang et al. [37] in a multicenter study with long-term follow-up. Mean lesion length was 44 mm and there was a non-significantly higher number of chronic total occlusions (CTOs) in the DCB arm (37.8 vs. 32.4%, *p* = 0.07). Follow-up angiography revealed similar diameter stenosis between the groups, while LLL was lower in the DCB arm. At 3 years, MACE and TLR rates were similar between the groups. Additionally, a DCB strategy proved once again safe, as no thrombotic events occurred in this group.

Diabetes mellitus (DM) represents one of the main risk factors for diffuse coronary lesions in small diameter vessels, consequently leading to increased risk of stent failure. It is easily understandable why DCB could represent an interesting alternative for these patients. Pan et al. [38] compared the outcomes of DCB-treated patients with or without DM using propensity analysis. In almost one-third of patients there was diffuse CAD. Compared to patients without DM, DM patients experienced higher TLF and TLR rates, but with a similar risk of MACE or any revascularization.

## 6. DCB in Left Main (LM)

The two largest RCTs to date comparing PCI with DES vs. CABG for LM revascularization offer somewhat conflicting results. On one hand, the 5-year follow-up of the EXCEL trial [39] reports similar rates of the primary composite of death, stroke, and MI but increased all-cause mortality following PCI. In contrast, the NOBLE trial [40] found that PCI was associated with inferior clinical outcomes at 5 years, mainly driven by higher rates of non-procedural MI and repeat revascularization. Nonetheless, even with intravascular imaging guidance and an optimal PCI result, a great source of repeat PCI in LM stenting is represented by ostial side-branch (SB) in-stent restenosis; thus, DCB could offer a promising alternative in this regard as part of a hybrid DES in main vessel DCB for ostial SB treatment.

Erdogan et al. [41] conducted a small prospective proof-of-concept study demonstrating that DCB for LM SB ostium is safe and effective at short-term follow-up. In a larger retrospective study [42], Liu et al. found a hybrid strategy of DCB in SB and DES in main vessel was superior to a two-stent strategy in terms of LLL both at the SB ostium and at the LM. There was no difference, however, in the incidence of MACE at the 1-year follow-up between the two groups. Pan et al. [43] compared using propensity matching 199 LM patients treated with a hybrid DES-DCB strategy to 398 LM patients assigned to DES-only therapy (provisional or two-stent) during a longer follow-up period. The primary endpoint was TLF, a composite of cardiac death, target vessel MI, and clinically driven TLR. At 2 years, compared to the DES-only group, the hybrid group was associated with lower TLF and TLR rates, but with similar death and stent thrombosis rates. What is more, SB LLL was lower in the hybrid group.

With these promising results in mind, some authors proposed a full-DCB strategy for de novo LM lesions. In a study on 66 patients (half of them with ACS), LM-PCI with a DCB-only strategy proved safe, and if lesion preparation was performed accordingly, it led to acceptable MACE (21.2%) and TLR (1.9%) rates at 1 year [44]. The SPARTAN-LMS study [45] compared DCB only vs. DES only for the treatment of LM stenosis. During a median follow-up of 33 months, there was a similar incidence of cardiac death, target vessel MI, or TLR between the two groups. Another retrospective study including patients with LM disease [46] found smaller LLL in the distal main vessel when comparing DCB only vs. DES only (−0.05 vs. 0.16 mm, *p* = 0.001), but no significant differences in ostial SB or proximal main vessel. Kitani et al. [47] performed directional atherectomy followed by DCB-PCI of LM lesions and found low TLR and cardiac mortality rates at long-term follow-up (5–5.9% and 0.8%, respectively) and reported no target vessel MI.

## 7. DCB in Acute Coronary Syndromes

ACS represents one clinical scenario that continues to be associated with higher restenosis and stent thrombosis rates mainly due to suboptimal stent sizing, apposition, and expansion, given the large thrombus burden and associated vasospasm. Thus, avoiding a permanent metal implant by using a DCB that has the potential of delivering an antiproliferative substance directly to the vulnerable plaque seems advantageous. Careful consideration should be given to the mechanism of plaque complication, as optical coherence tomography (OCT) imaging shows worse TLR with DCB-treated calcified nodules as compared to plaque rupture or plaque erosion. Additionally, post-PCI residual thrombus predicts TLR, especially in patients with plaque rupture [48].

The PEPCAD NSTEMI RCT [49] demonstrated the non-inferiority of DCB compared to DES in terms of TLF and MACE rate. For STEMI patients, two studies are noteworthy. In the REVELATION RCT [50], DCBs were non-inferior to DESs regarding the 9-month fractional flow reserve value. In a large propensity-matched analysis [51] including 1139 STEMI patients with de novo lesions, DCBs were similar to DESs in terms of all-cause mortality (10.8 vs. 9.0%, *p* = 0.18) and unplanned TLR during 3 years of follow-up.

In a dedicated sub-analysis of the BASKET-SMALL 2 RCT [52], there was a significant interaction between clinical presentation and treatment at 1 year, with lower rates of cardiac death (*p* for interaction, 0.04) and non-fatal MI seen in ACS patients treated with DCB (*p* for interaction, 0.01). These results, however, were not sustained at the long-term follow-up.

A recent meta-analysis [53] including 67% ACS patients treated with a DCB strategy found no significant differences in the incidence of TLR between the DCB and DES groups (4.3 vs. 6.9%, OR 0.71, 95% CI 0.5–1.0, *p* = 0.059) over a pooled follow-up of 25.8 ± 2.7 months. Abdelaziz et al. [54] conducted a meta-analysis of 13 studies specifically comparing DCB vs. DES in the setting of ACS. The pooled OR showed that DCBs were non-inferior to DESs in terms of MACE and, what is more, when MACE was defined as a composite of only cardiac death, MI, and TLR, DCBs outperformed DESs (OR 0.5, 95% CI 0.28–0.9, *p* =  0.02). Non-inferiority was also observed in the case of cardiac death, MI, and LLL.

## 8. Dcb in High-Bleeding-Risk (Hbr) Patients

Bleeding following PCI represents one important matter in current-day practice, especially as PCI is performed on an increasingly older population at high bleeding risk (HBR) and is associated with a significant rise in mortality [55]. As DCB-PCI would avoid a permanent prothrombotic metallic coronary implant, shorter DAPT duration or even omitting the second antiplatelet drug seems feasible. It should be noted, however, that in recent years, reducing the DAPT duration in HBR patients treated with DES has been intensively studied as well. In the MASTER DAPT trial, for example, Valgimigli et al. reported the non-inferiority of one-month DAPT versus standard 3-month DAPT in terms of net adverse clinical events and major adverse cardiac or cerebral events, with the advantage of a lower incidence of major or clinically relevant nonmajor bleeding (6.5% vs. 9.4%, difference, −2.82 percentage points; 95% CI, −4.40 to −1.24; *p* < 0.001 for superiority) [56]. Nevertheless, DAPT should be considered following an ACS to prevent further ischemic events.

The DEBUT RCT [57] compared DCB vs. BMS in the setting of HBR, excluding ST elevation MI (STEMI) patients. At 9 months, DCBs were superior to BMSs regarding the primary outcome, a composite of cardiac death, MI, and TLR (1 vs. 14%, *p* = 0.00034 for superiority). In the prespecified analysis of the BASKET-SMALL-2 RCT [58], 155 HBR patients treated with DCBs were identified. There were no differences regarding the 3-year MACE (HR: 1.16 [0.51–2.62]; *p* = 0.719) as well as the rate of major bleedings (4.5 vs. 3.4%) between the DCB and DES groups. Cancer patients represent a high-risk group where DCBs seem to offer an advantage over DESs, especially in the setting of ACS. Yang et al. [59] demonstrated that DCBs are associated with numerically lower rates of MACE and major bleedings compared to DESs in these patients.

Current European guidelines [60] tackling DAPT in HBR propose a DAPT duration of 1–3 to 6 months (class II recommendation) following DCB-PCI, depending on the clinical presentation, while The International DCB Consensus suggests 1-month DAPT based on expert opinion [22]. Interestingly, a single antiplatelet strategy seems feasible and safe, as seen in two retrospective studies [22,61]. Rasanen et al. [61] found acceptable rates of MACE, TLR, and significant bleedings (10.5%) in a cohort of 172 complex HBR patients treated with DCBs. Cortese and Serruys [62] found that HBR patients treated with a single antiplatelet had similar MACE (9 vs. 10%) and lower rates of significant bleedings (6 vs. 9%, *p* = 0.04) compared to a general DCB cohort treated with DAPT.

## 9. DCB in Chronic Total Occlusion

CTOs represent an important and challenging scenario, estimated to represent approximately 13–20% of CAD, with higher procedural risks and complications and worse long-term outcomes when compared to standard PCI [63]. As such, the possible benefits of DCB usage in these lesions have been intensely analyzed. A meta-analysis by Zhao Y et al. [64] showed that the treatment of CTOs with DCBs when compared to exiting data for DES usage in CTOs (DECISION CTO and DECISION COTRAIL) may provide better results with regard to MACE, while presenting similar TVR and TLR rates.

Wang X. et al. [65] obtained similar MACE rates at 3-year follow-up between CTOs treated with DES only vs. hybrid or DEB only, that is 12% with regard to restenosis and re-occlusion rates; the results were similar in the two groups, with rates of restenosis of around 20% at 1-year angiographic follow-up. Other studies for the use of DCBs in CTOs provided lower restenosis rates (11.8% and 17%, respectively) [66,67], albeit with shorter angiographic follow-up periods.

**Figure 4:**
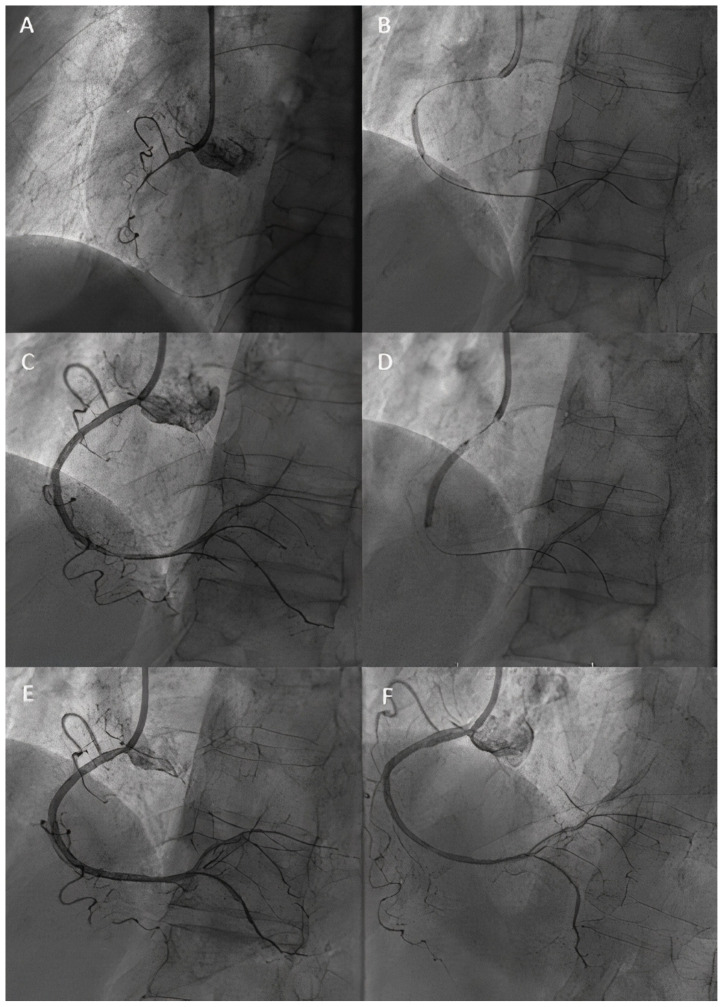
DCB in CTO lesions. (**A**) Diagnosis: CTO of RCA. (**B**) Lesion preparation. (**C**) Result after predilatation (**D**) DCB treatment of the CTO segment. (**E**) Result after DCB. Notice residual ostial stenosis. (**F**) Result after ostial stenting.

**Figure 5:**
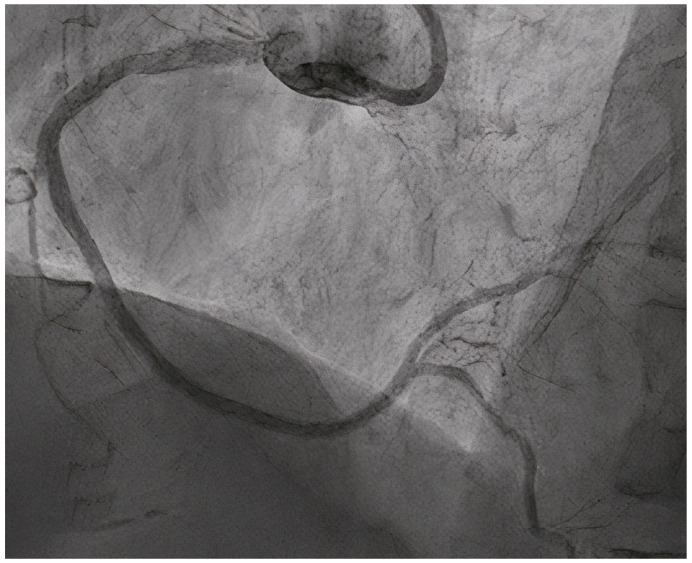
One-year follow-up of RCA CTO treated by a hybrid approach, showing important LLL in the DCB-treated segment.

A significant advantage of DCBs over DESs in de novo CTOs might be the lower risk of sub expansion and higher rates of LLE (late lumen enlargement). Existing data suggest rates of LLE between 48 and 69% [64,65,68] for de novo CTO lesions treated with DCBs. This in turn leads to lower rates of LLL, defined as the difference in MLD after the procedure and at angiographic follow-up [65]. The possibility of positive vessel remodeling post-recanalization can be explained by the fact that, in time, the previously hypo-perfused coronary artery responds to vasomotor reflexes and reacquires its original size, which might not be possible in the case of an under-sized DES during PCI [69,70]. Figure 4 illustrates the excellent angiographic result of a right coronary artery CTO lesion treated by a hybrid approach (DCB for the CTO segment + DES for the ostial lesion), with important late lumen enlargement seen after one year, as can be seen in Figure 5. As previously explained, the importance of intravascular imaging, in order to better understand the plaque morphology, real lumen size, and therefore choose the appropriate predilatation tools, as well as aggressive lesion preparation in order to obtain an adequate acute lumen gain before final DCB treatment, becomes vital, as in this particular setting, it is usually extremely difficult to determine the correct size of the vessel, as well as to understand the underlaying mechanism behind the total occlusion.

In conclusion, available data suggest that DCBs are a viable non-inferior alternative to the classical DES approach, both in terms of MACE and restenosis or reocclusion rate. As limitations we should point out the lack of large scale multicenter randomized control studies.

## 10. DCB in Bifurcation Lesions

Bifurcation lesions constitute an area of high interest when it comes to the use of DCBs. This can be performed either as a DEB stand-alone procedure—for the main or side branch of the bifurcation or for both branches—or as a hybrid technique for the side branch of a bifurcation. The appeal of DCB in side branch is understandable, avoiding an unnecessary two-stent technique but also not limiting the treatment to POBA.

The hybrid treatment of bifurcation lesions (DES in main branch and DEB in side branch) demonstrates a high rate of procedural success, and seems to yield comparable long-term results to two-stent techniques, although further studies are required [71]. In a meta-analysis performed by Corballis NH et al. [72], factoring for LLL, DCB proved superior to POBA for side-branch treatment. Another meta-analysis by Megaly M et al. [73] demonstrated lower rates of LLL in the DCB group compared to POBA for side branch treatment. However, there was no significant difference in MACE between the two groups at 15 months [73]. While all of these studies only compared DCB to POBA for the treatment of side branch, in a recently published pilot study, Ke D et al. randomized 60 patients with true bifurcation lesions to be treated by a DCB-based strategy or DST-based strategy, with one-year LLL and MACE representing the primary endpoints [74]. With a high rate of cutting/scoring balloon usage for lesion preparation in both arms, the authors reported significantly lower 1-year LLL in the DCB-based group, without any differences in the MACE rate, however [74].

Regarding usage of DCB for main branch treatment, the most common case is for Medina 0-1-0/0-1-1. Kleber FX. et al. [75] proved that DCB is superior to POBA when accounting for LLL and restenosis in cases where the proximal part of the main branch was not involved. Other studies also suggest that the DCB-only strategy is efficient and safe for selected bifurcation lesions, with the advantage of shorter DAPT duration treatment [76,77].

**Figure 6:**
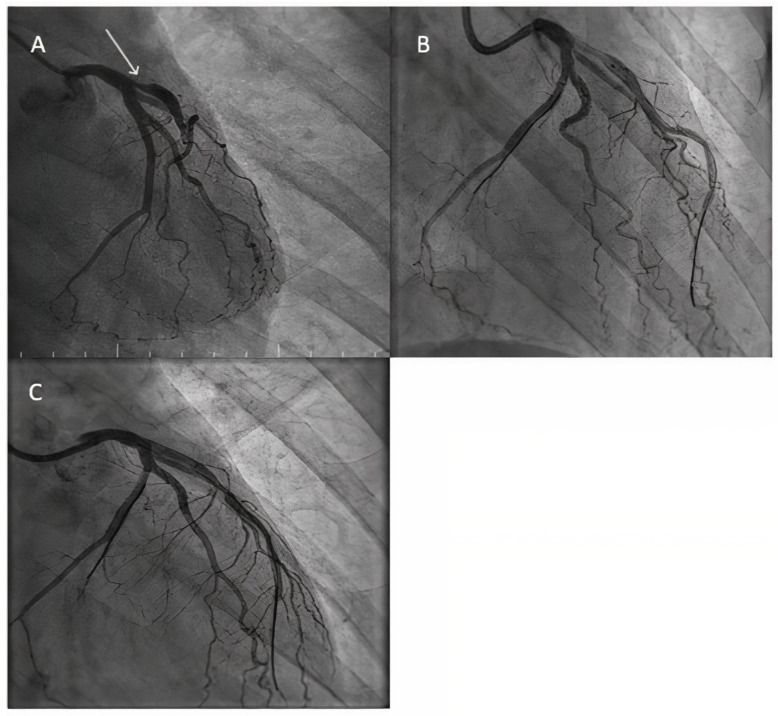
Ostial LAD stenosis treated by an LM-LAD DCB approach. (**A**) Angiographic aspect of a severe ostial LAD stenosis. (**B**) The aspect of the vessel after predilatation. (**C**) Final angiographic result.

Figure 6 illustrates a modern approach of a Medina 0-0-1 left main bifurcation in a very young patient (29 years old), with excellent angiographic results, thus emphasizing the advantage of DCB, as this type of bifurcation lesion could be solved without involving the left main and stenting it.

Table 2 summarizes the performance of DCB in different angiographic settings, as reported by the major studies.

## 11. Conclusions

Drug-eluting stents remain the gold standard in treating most of the coronary lesions. However, long-term risks, such as in-stent restenosis and stent thrombosis, are still present, despite continuous technological improvements. For this purpose, drug-eluting balloons have emerged as promising tools in reducing these limitations, with recent data emphasizing their potential to improve long-term results alone or in combination with stents, especially in complex lesions. With results from large, randomized trials still awaited, we can now assume that a cautious selection of patients and lesions, alongside the use of optimal lesion preparation tools and imaging. represent key steps in optimizing PCI long-term results.

## Figures and Tables

**Table 1:** The performance of DCB in small vessel disease.

Study	Population	Endpoint	Results
**PICCOLETO II [15]**	232 patients (DCB vs. DES)	6-month LLL	0.04 vs. 0.17 mm; *p* = 0.001 for non-inferiority; *p* = 0.03 for superiority **3-year follow-up:** **MACE:** 20.8% vs. 10.8% [*p* = 0.046] **Acute vessel occlusion:** 4% vs. 0% [*p* = 0.042] **All cause death:** 4% vs. 3.9%; *p* = 0.98 **MI:** 6.9% vs. 2%; *p* = 0.14 **Cardiac death:** 1% vs. 1.9%; *p* = 0.56 **TLR:** 14.8% vs. 8.8%; *p* = 0.18
**BASKET SMALL 2 [17]**	758 patients (DCB vs. DES)	MACE	**3-year follow-up:** **stent thrombosis (DCB vs. DES):** Kaplan–Meier estimate 1% vs. 2%; HR 0.33, 95% CI 0.07–1.64; *p* = 0.18) **major bleedings (DCB vs. DES):** Kaplan–Meier estimate 2% vs. 4%, HR 0.43, 95% CI 0.17–1.13; *p* = 0.088
**RESTORE SVD [18]**	230 patients (DCB vs. DES)	DS %	**DS%:** (29.6 ± 2.0% versus 24.1 ± 2.0%; the 1-sided 97.5% upper confidence limit of the difference was 10.9%) **TLF (1 year):** 4.4% vs. 2.6%, *p* = 0.72
**ANDROMEDA [19]**	1154 patients (1360 lesions)	n/a	**MACE (DCB vs. DES):** hazard ratio (HR) 0.67, 95% confidence interval (CI) 0.47–0.96

DCB: drug-coated balloon; DS%: in-segment percentage diameter stenosis; LLL: late lumen loss; MACEs: major adverse cardiac events; MI: myocardial infarction; n/a: not applicable; TLF: target lesion failure; TLR: target lesion revascularization.

**Table 2:** DCB vs. DES in different angiographic settings.

**Study**	**Angiographic Scenario**	**Main Findings**
**Isar-Desire 3 [8,9]**	in-stent restenosis	**3-year follow-up:** **Lower risk of death for DCB:** HR: 0.38, 95% CI: 0.17 to 0.87; *p* = 0.02 **10-year follow-up:** composite of CD, TVMI, TLT, and TLR: 72% (POBA) vs. 55.9% (PCB) vs. 62.4% (DES), *p* < 0.001
**Zhu Y et al. [13]**	in-stent restenosis	**lower TLR** rates for DES implantation (risk ratio = 1.53, 95% CI 1.15–2.04, *p* = 0.003)
**PICCOLETO II [15]**	small vessel disease	lower **LLL** rates for DCB angioplasty **similar hard clinical endpoints at 3-year follow-up** (Table 1)
**BASKET SMALL 2 [17]**	small vessel disease	**similar MACE** at 3-year follow-up
**RESTORE SVD [18]**	small vessel disease	similar TLF and DS% rates at 1-year follow-up
**ANDROMEDA [19]**	small vessel disease	**similar TLF rates** at 3-year follow-up, but with a **lower MACE rate** compared with DES
**Yu X et al. [26]**	de novo large vessel disease	**similar 12-month MACE rates** (2.44% vs. 6.33%; *p* = 0.22)
**Sun B et al. [28]**	de novo large vessel disease	**lower LLL** in the DCB group (MD −0.13, 95% CI −0.22 to −0.05, *p* = 0.003, I2 = 60%)
**Costopoulos et al. [33]**	hybrid approach	**similar MACE** (20.8 vs. 22.7%; *p* = 0.74) **similar TLR** (9.6 vs. 9.3%; *p* = 0.84)
**Yang et al. [37]**	hybrid approach	**similar DS%** (31.96 ± 17.21 vs. 30.67 ± 18.80%, *p* = 0.622) **lower LLL in the DCB group** (0.06 ± 0.61 vs. 0.41 ± 0.64 mm, *p* < 0.001) **At 3-year follow-up:** **similar MACE** (11.3 vs. 13.7%, *p* = 0.32) **and TLR** (7.3 vs. 8.3%; *p* = 0.63)
**Pan L et al. [38]**	hybrid approach diabetic vs. non-diabetic patients	**higher TLF** (5.36 vs. 2.77%, *p* = 0.025) **and TLR** (5.36 vs. 2.77%, *p* = 0.025) rates in diabetic patients **similar risk of MACE** (OR: 1.580, 95% CI: 0.912–2.735) **or any revascularization** (OR: 1.534, 95% CI: 0.983–2.393; *p* = 0.058).
**Liu et al. [42]**	left main hybrid approach vs. DES	**lower LLL for a hybrid strategy in the SB** (−0.17 vs. 0.43 mm; *p* < 0.001), **as well as in the LM stem** (0.09 vs. 0.17 mm; *p* = 0.03)
**Pan et al. [43]**	left main	**lower TLF** (7.56 vs. 14.36%, *p* = 0.025) **and TLR** (2.91 vs. 9.42%, *p* = 0.007) **lower LLL** (0.13 ± 0.42 vs. 0.42 ± 0.62 mm, *p* < 0.001).
**SPARTAN-LMS [45]**	left main	similar **CD** (4.9 vs. 6.5%, *p* = 0.786), **TVMI** (0 vs. 6.5%) or **TLR** (7.3 vs. 8.3%, *p* = 0.86)
**PEPCAD NSTEMI [49]**	acute coronary syndromes	**similar TLF** (3.8 vs. 6.6%, *p* = 0.53) and **MACE rates** (6.7 vs. 14.2%, *p* = 0.11)
**REVELATION Trial [50]**	acute coronary syndromes	**similar 9-month FFR** (0.92 ± 0.05 vs. 0.91 ± 0.06, *p* = 0.27)
**Abdelaziz A [54]**	acute coronary syndromes	**similar MACE** (OR 0.89, 95% CI 0.57–1.4, *p* = 0.63) **similar rates of CD** (OR 0.59, 95% CI 0.22–1.56, *p* = 0.29), **MI** (OR 0.88, 95% CI 0.34–2.29, *p* = 0.79) **and LLL** (MD = −0.6, 95% CI −0.3–0.19, *p* = 0.64)
**Rasanen et al. [61]**	high-bleeding-risk patients	**low rates of MACE** (4.1% stable CAD; 12.1% ACS), **TLR** (0% stable CAD; 3.0% ACS)
**Zhao Y et al. [64]**	chronic total occlusion	**lower MACE** (13% vs. 21.5%) **similar TVR** (7.1% vs. 7.9%) and **TLR** rates
**Ke D et al. [74]**	bifurcation lesions	**lower LLL** vs. DES (main-vessel: 0.05 ± 0.24 mm vs. 0.25 ± 0.35 mm, *p* = 0.013; side-branch: –0.02 ± 0.19 mm vs. 0.11 ± 0.15 mm, *p* = 0.005)

CD: cardiac death; DCB: drug-coated balloon; DES: drug-eluting stent; DS%: in-segment percentage diameter stenosis; LLL: late lumen loss; LM: left main; MACEs: major adverse cardiac events; MI: myocardial infarction; POBA: plain old balloon angioplasty; SB: side branch; TVMI: target vessel myocardial infarction; TLF: target lesion failure; TLT: target lesion thrombosis; TLR: target lesion revascularization.
